# Clinical Features of Dermatomyositis Associated with Myositis-Specific Antibodies in Moroccan Patients

**DOI:** 10.3390/clinpract15020031

**Published:** 2025-02-06

**Authors:** Milouda Chihi, Leila Barakat, Fatima Zahra Benhayoun, Abire Allaoui, Samy Housbane, Mina Moudatir, Fouzia Hali, Ahmed Aziz Bousfiha, Jalila El Bakkouri

**Affiliations:** 1 Laboratory of Inflammation Clinical Immunology and Allergy(LICIA), Faculty of Medicine and Pharmacy of Casablanca, Hassan II University, Casablanca 20250, Morocco; milouda.chihi-etu@etu.univh2c.ma (M.C.); minamoudatir@gmail.com (M.M.);; 2 Internal Medicine Department, Ibn Rochd University Hospital, Casablanca 20100, Morocco; 3 Dermatology Department, Ibn Rochd University Hospital, Casablanca 20100, Morocco; 4 Internal Medicine Department, Cheikh Khalifa International University Hospital, Casablanca 82403, Morocco; 5 Immunopathology-Immunomonitoring-Immunotherapy Laboratory, Mohammed VI University of Health and Sciences (UM6SS), Casablanca 82403, Morocco; 6 Medical Informatics Laboratory, Faculty of Medicine and Pharmacyof Casablanca, Hassan II University, Casablanca 20250, Morocco; 7 Department of Pediatric Infectious Diseases and Clinical Immunology, Abderrahim Harouchi Mother-Child Hospital, Ibn Rochd University Hospital, Casablanca 20100, Morocco; 8 Laboratory of Immunology, Ibn Rochd University Hospital, Casablanca 20100, Morocco

**Keywords:** antibodies, clinical features, dermatomyositis, myositis

## Abstract

**Background/Objectives:** Dermatomyositis (DM) is a rare idiopathic inflammatory myopathy characterized by muscle weakness and typical cutaneous rash. Dermatomyositis-specific antibodies, such as anti-TIF1γ, anti-SAE, anti-Mi2, anti-MDA5, and anti-NXP2, have been associated with specific clinical phenotypes. Our study aimed to describe the clinical profile of Moroccan patients with DM and clinical associations with myositis-specific antibodies. **Methods:** We recruited 54 adult patients with DM according to the Bohan and Peter criteria, admitted to the internal medicine and dermatology departments of the University Hospital Center Ibn Rochd of Casablanca from January 2020 to December 2023. Testing for myositis-specific autoantibodies (MSAs) was conducted using an Immunodot assay. Statistical analysis was performed using the Chi-square test. **Results:** Among our patients, 74% were female. The mean age of the patients at the time of diagnosis was 45.8 years (±12.95 years). The main clinical manifestations were a V-neck sign (70.4%), myalgia (70.4%), Gottron’s papules (68.5%), heliotrope rash (63%), arthritis/arthralgia (48.1%), proximal muscle weakness (68.5%), periungual erythema (46.3%), and dysphagia (59.3%). Of the 54 patients, 37 (68.5%) showed dermatomyositis-specific antibody positivity. The most frequently found autoantibody was anti-Mi2 (22.2%), followed by anti-TIF1γ (14.8%), anti-NXP2 (9.2%), anti-MDA5 (7.4%), and anti-SAE (7.4%). The association between clinical manifestations and MSAs showed that anti-TIF1γ antibodies were associated with the V-neck sign (*p* < 0.05), and the MSA-negative group was protected from periungual erythema (*p* < 0.05). No other significant association was found. **Conclusions:** This study shows the autoantibody profile of Moroccan patients with DM and the associations of MSAs with clinical manifestations.

## 1. Introduction

Dermatomyositis (DM) is a rare autoimmune disease characterized by a typical skin rash and muscle weakness [1]. The first diagnostic and classification criteria of DM were established by Bohan and Peter in 1975, including five main criteria: symmetric proximal muscle weakness, elevated muscle enzymes, myopathic changes in electromyography (EMG), characteristic abnormalities in muscle biopsy, and a skin rash typical of DM [2]. In 2017, the EULAR/ACR (European League Against Rheumatism/American College of Rheumatology) classification criteria for Idiopathic Inflammatory Myopathies (IIM) established a score for the probability of having DM based on easily measurable characteristics, such as clinical manifestations and elevation inmuscle enzymes, with or without the inclusion of muscle biopsies. The total score determines whether a patient can be classified as having definite DM or probable DM [3].

Autoantibodies can be categorized into two groups: myositis-associated antibodies (MAAs), usually found in other rheumatological diseases, and myositis-specific antibodies (MSAs), found exclusively in inflammatory myopathies. The most relevant MSAs for DM are anti-Mi2 (against nucleosome remodeling and histone deacetylase protein complex), anti-TIF1γ(against transcriptional intermediary factor 1γ), anti-NXP2 (against nuclear matrix protein 2), anti-SAE (against small ubiquitin-like modifier activating enzyme), and anti-MDA5 (against melanoma differentiation-associated gene 5). Each MSA is associated with a distinct clinical subset. Anti-Mi2 antibodies are often associated with severe muscle involvement and typical cutaneous manifestations, while extramuscular involvement is less common [4,5]. Anti-NXP2 antibodies are described as a marker of juvenile DM and are associated with calcinosis and dysphagia [6,7].

Anti-MDA5 antibodies are associated with lung involvement, rapidly progressive interstitial lung disease (RP-ILD), and skin ulcerations [8,9]. Anti-SAE antibodies are associated with dysphagia and cutaneous manifestations, while anti-TIF1 γ antibodies are highly associated with malignancy and severe skin manifestations [10].

The utility of MSAs in DM patients is crucial, as these autoantibodies are specific to DM. MSAs can confirm the diagnosis in atypical cases of DM, such as DM sine dermatitis and amyopathic DM, thereby reducing the risk of a misdiagnosis. Additionally, MSAs are associated with distinct clinical phenotypes and can predict the disease course and response to treatment. The autoantibody profile and the association between MSAs and clinical manifestations vary by ethnicity [11].

However, studies on the association between clinical manifestations and MSAs in Moroccan patients are lacking. Therefore, the present study aimed to describe the clinical profile of Moroccan patients with DM, and analyze the possible associations between MSAs and clinical manifestations.

## 2. Materials and Methods

### 2.1. Study Population

We recruited 54 adult Moroccan patients diagnosed with DM according to the Bohan and Peter criteria from the Dermatology and Internal Medicine departments of University Hospital Center Ibn Rochd Casablanca over 3 years, from January 2020 to December 2023. The following data were collected:

- Demographics: gender, age at diagnosis, age at disease onset, and ethnicity.
- Clinical manifestations: skin involvement (Gottron’s papules, heliotrope rash, V-neck sign, shawl sign, holster sign, alopecia, poikiloderma, skin ulcers, necrotic ulcers, and periungual erythema), systemic involvement (dysphagia, Raynaud’s phenomenon, arthralgia, and ILD), and muscle involvement (myalgia and proximal muscle weakness).
- Muscle biopsy, skin biopsy, EMG, and muscle enzyme testing the following: creatine kinase (CK) andlactate dehydrogenase (LDH);
- Screening for cancer.

### 2.2. Patient Consent and Ethics

The institutional ethics committee of the University Hospital Center Ibn Rochd approved this study (n. SRSI/105/n13/2023). Written informed consent was obtained from all participants.

### 2.3. Antinuclear Antibody Testing

Serum samples were collected and stored at −20 °C until use. Testing for antinuclear antibodies was performed using Indirect Immuno Fluorescence (IIF) on HEp-2 cells, following the manufacturer’s instructions (EUROIMMUN, Medizinische Labordiagnostika).

### 2.4. Inflammatory Myopathies Auto-Antibodies Testing

The identification of inflammatory myopathies auto-antibodies was performed by Immunodot using EUROLINE Autoimmune inflammatory myopathies 16 Ag IgG Dot (Lot: DL 1530-1601-4 G): Mi-2α, Mi-2β, TIF1 γ, MDA5, NXP2, SAE1, Ku, PM-Scl100, PM-Scl75, Jo-1, SRP, PL-7, PL-12, EJ, OJ and Ro-52.

### 2.5. Statistical Analysis

The Chi-square test assessed the association between MSAs and clinical manifestations. Fisher’s exact test was used when more than 20% of cells had an expected count of less than 5 in a 2 × 2 contingency table. Statistical significance was set at *p* < 0.05. All statistical analyses were conducted using IBM SPSS Statistics version 21.0.0.0.

## 3. Results

### 3.1. Demographics and Clinical Features of DM Patients

Fifty-four patients with DM were enrolled in this study, and their general characteristics are presented in Table 1.

The mean age at diagnosis was 45.8 years (standard deviation, ±12.95 years), and the mean age of the patients at disease onset was 45.6 years (standard deviation, ±12.93 years).

We noted a female predominance, as females represented 74% of our cohort.

In our cohort, three patients were diagnosed with amyopathic dermatomyositis (ADM) and four with dermatomyositis sine dermatitis (DMSD). Among the ADM cases, one patient tested positive for anti-MDA5 antibodies, one was negative for myositis-specific autoantibodies (MSAs), and one tested positive for anti-Mi2 antibodies. Of the four DMSD cases, two were positive for anti-NXP2 antibodies, one for anti-MDA5 antibodies, and one for anti-Mi2 antibodies.

Regarding malignancy, six patients had paraneoplastic DM; three patients had breast cancer, two patients lung cancer, and one patient nasopharyngeal carcinoma.

**Figure 1:**
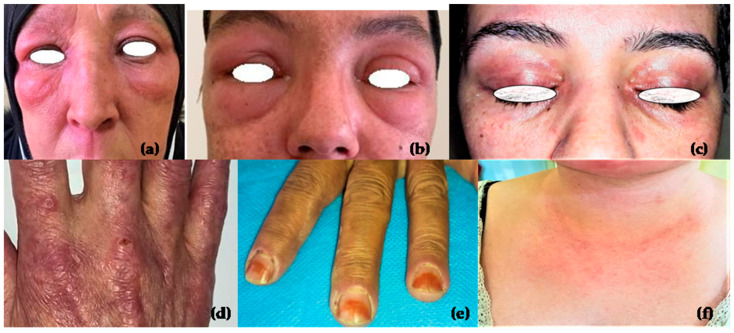
Cutaneous manifestations of DM patients: (**a**,**b**) heliotrope rash with edema; (**c**) heliotrope rash; (**d**) Gottron’s papules; (**e**) periungual erythema; (**f**) V-neck sign.

In our study, the most common clinical manifestations were myalgia (70.4%), a V-neck sign (70.4%), Gottron’s papules (68.5%), heliotrope rash (63%), and dysphagia (59.3%). Cutaneous manifestations are presented in Figure 1.

### 3.2. Paraclinical and Histopathological Findings

The EMG findings were compatible with DM in 42 out of 43 patients. Skin biopsy was performed in 15 patients, of which 10 were compatible with DM. Muscle biopsy was compatible with DM in 11 of 17 patients.

Muscle biopsy: perifascicular atrophy with lymphocytic infiltrates was the most consistent feature in five biopsies. Two of these five also showed necrosis. Focal inflammatory infiltrates without necrosis were seen in three biopsies. Necrotic and regenerating myocytes were observed in one biopsy. Micro-infarctions, characterized, were identified in one biopsy. A biopsy showed nonspecific results with few regenerating fibers. Inflammation was significant in six biopsies, five perivascular and one endomysial.

Skin biopsy: the most frequent histopathological finding was interface dermatitis, seen in six biopsies. Mucin deposition was observed in four biopsies. The perivascular lymphocytic infiltrate was seen in three biopsies. One biopsy showed keratinocyte necroses, vascular necrosis, and epithelial edema.

Testing for CK and LDH was performed for all patients. Fifty patients had elevated levels of CK and LDH, while four were within the normal range. EMG revealed signs of myopathy in three of the four patients with normal levels of CK and LDH. Table 1 summarizes the patient’s general characteristics.

### 3.3. Detection of ANA and MSAs

In our study, all patients were tested for ANA and MSAs; 35 (64.8%) tested positive for ANA and 37 (68.5%) tested positive for at least one MSA.

Out of the 35 patients who tested positive for ANA, 11 had a low titer (1:160), 11 had a titer of 1:320, 6 had a titer of 1:640, and 7 had a titer of 1:1280.

**Figure 2:**
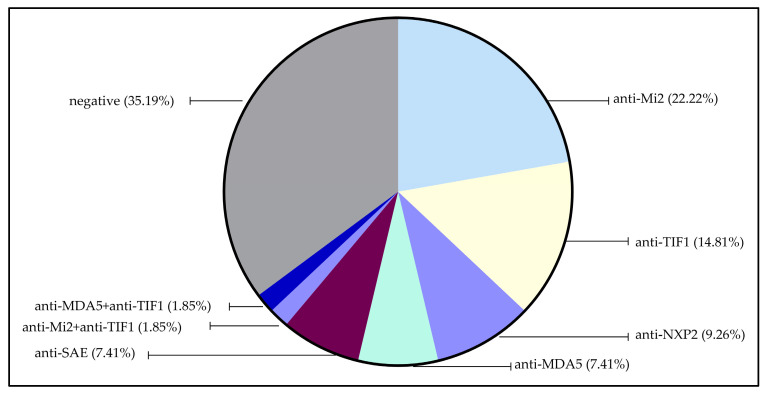
Prevalence of the detected MSAs in our cohort.

Regarding the ANA patterns, 18 patients had a speckled pattern, 4 had a homogenous+speckled pattern, 3 had a cytoplasmic pattern, 2 had a homogenous pattern, 1 had a nuclear dots pattern, and 1 had a Golgi pattern. The ANA patterns of our patients are presented in Figure 2.

The most detected MSA in our study was anti-Mi (22.22%), followed by anti-TIF1γ (14.81%), anti-NXP2 (9.26%), anti-MDA5 (7.41%), and anti-SAE (7.41).

There were no identifiable MSAs in 19 patients (35.19% of the cohort).

The coexistence of two MSAs was observed in two patients: one with anti-TIF1γ and anti-MDA5 antibodies, and another with anti-Mi2 and anti-TIF1γ antibodies (Figure 2).

### 3.4. Association of MSAs with Clinical Manifestations

In Table 2, we analyzed the association of the clinical manifestations of DM, including skin rash, proximal muscle weakness, arthralgia, ILD, malignancy, dysphagia, and Raynaud’s phenomenon with the type of MSAs.

The V-neck sign was associated with anti-TIF1γ (*p* < 0.05). The MSA-negative group was associated with periungual erythema (*p* < 0.05).

We found no other significant associations between clinical manifestations and MSAs (*p* > 0.05). The results of the associations between clinical features and MSAs are shown in Table 2.

## 4. Discussion

This study investigated the clinical and autoantibodies profiles of Moroccan adult patients diagnosed with dermatomyositis (DM) based on the Bohan and Peter criteria [2]. A total of 54 patients were enrolled, with the findings reflecting the heterogeneity of DM across clinical presentations and autoantibody subgroups. Our cohort demonstrated a significant female predominance (74%), consistent with other autoimmune diseases. The mean age at onset was 45.6 years (±12.93 years), comparable to the 47.3 years reported in a Brazilian cohort [12]. Antinuclear antibodies (ANAs) were detected in 64.8% of our patients, a proportion lower than the 80–90% reported in the literature [13]. Myositis-specific antibodies (MSAs), the biomarkers for diagnosis, classification, and prognosis, were identified in 68.5% of our patients, aligning with the 71.4% and 63% reported by Le et al. [14] and Babu et al., respectively [15]. Anti-Mi2 was the most frequent antibody in our study (22.22%), followed by anti-TIF1γ (14.81%), anti-NXP2 (9.26%), anti-MDA5 (7.41%), and anti-SAE (7.41%). Notably, 35.19% of patients were negative for MSAs, reflecting the heterogeneity of DM. Comparatively, Le et al. reported anti-TIF1γ (26.5%), anti-Mi2 (14.3%), anti-MDA5 (16.3%), anti-SAE (10.2%), and anti-NXP2 (4.1%) [14], while in Brazil, anti-Mi2 and anti-MDA5 were equally prevalent (11% each), followed by anti-TIF1γ (7.7%), anti-SAE (6.6%), and anti-NXP2 (5.5%) [12]. Double-positive MSAs are rare; in our cohort, one patient had anti-TIF1γ and anti-MDA5, and another had anti-TIF1γ and anti-Mi2, consistent with previous reports. Ito et al. reported a rare case of classic DM with both anti-NXP2 and anti-Mi2 antibodies, where the patient presented with a typical skin rash and muscle weakness without dysphagia, ILD, calcinosis, or malignancy [16]. Similarly, another study found double-positive MSAs (anti-MDA5 and anti-Mi2 antibodies) [17].

The hallmark cutaneous features of DM, including Gottron’s papules, heliotrope rash, shawl sign, a V-neck sign, and periungual erythema, were prominent in our cohort. Gottron’s papules were the most common, observed in 68.5% of patients, higher than the rates reported by Le et al. (50.9%) and Alenzi et al. (56.3%) [14,18], but lower than the 96.7% reported in Brazil [12]. Other skin abnormalities, such as skin ulcers, are associated with anti-MDA5 antibodies and indicate a poor prognosis. In our cohort, skin ulcers and necrotic ulcers were observed in 27.8% and 9.3% of patients, respectively. Skin biopsy findings varied across autoantibody subgroups; in anti-TIF1γ patients, biopsies showed interface dermatitis, mucin deposition, atrophic epidermis with hyperorthokeratosis, and mild perivascular lymphocytic infiltration; anti-Mi2 patients presented with interface dermatitis; anti-NXP2 patients exhibited keratinocyte necrosis, and vascular necrosis; and anti-MDA5 patients displayed perivascular dermatitis with interstitial mucin deposition. Vasculopathy was significantly associated with anti-MDA5 cases [19]. A study by Shakshouk et al. reported high frequencies of interface dermatitis (95%) and perivascular lymphocytic infiltrate (71%) in DM [19]. In the literature, anti-Mi2 antibodies are associated with characteristic skin changes, including erythema in photoexposed areas, heliotrope rash, and Gottron’s papules. Regarding muscle involvement, anti-Mi2-positive patients tend to have severe muscle damage [1]. A Chinese study involving 264 DM patients showed that anti-MDA5 was associated with skin ulcerations in both typical DM and clinically amyopathic DM groups [20]. Similarly, a study by Truzzi et al. showed that anti-MDA5 was related to digital ulcers, arthritis, vasculitis, and male gender [12]. Skin involvement is prominent in patients with anti-TIF1γ. In our cohort, patients with anti-TIF1γ had severe cutaneous manifestations with a significant association with a V-neck sign (*p*-value = 0.024). We also found that patients with no MSAs had a protective effect against periungual erythema (*p*-value = 0.03). Anti-NXP2 antibodies (9.3%) were commonly associated with Gottron’s papules, heliotrope rash, erythroedema, a V-neck sign, arthralgia, and dysphagia in our cohort. However, some atypical skin manifestations may occur, such as Degos-like skin lesions [21]. While calcinosis is frequently associated with anti-NXP2 [22], it was only observed in one patient who tested positive for anti-MDA5 antibodies.

Muscle involvement was prominent, with 68.5% of patients reporting proximal muscle weakness and 70.4% experiencing myalgia, rates comparable to the findings of Le et al. (59.7% and 79.2%, respectively) [14]. Elevated serum muscle enzymes such as creatine kinase (CK) and lactate dehydrogenase (LDH) are common due to muscle damage. However, 10 to 26% of patients present with normal CK levels, particularly those with amyopathic DM [23]. Several factors may explain this, including minimal or absent muscle involvement, early-stage disease, focal muscle inflammation, or individual variability in enzyme release, accenting the need for electromyography or muscle biopsy in such cases [24]. In our study, four patients (7.4%) had normal levels of CK and LDH; they presented with a typical skin rash of DM, including Gottron’s papules, periungual erythema, and a V-neck sign. Regarding extracutaneous involvement, no patients presented with ILD, and one patient had dysphagia. A study by Soontrapa et al. [23] found that DM patients with normal CK levels weresignificantly less likely to have cutaneous involvement. Muscle biopsy findings revealed distinct patterns across autoantibody subgroups; in anti-Mi2 patients, biopsies showed necrosis, perifascicular atrophy, inflammation, and focal inflammatory infiltrates without necrosis. Gudipati et al. reported higher frequencies of muscle fiber necrosis in anti-Mi2 compared to the other auto-antibodies groups [4]. Anti-MDA5 patients exhibited focal necrotic myocytes with endomysial inflammation or nonspecific findings with regenerating fibers. Allenbach et al. compared the histopathological findings between anti-MDA5 and classic DM, and identified that muscle biopsy specimens did not present the classical features of perifascicular atrophy [25]. Anti-NXP2 patients showed micro-infarctions or perifascicular atrophy with perivascular inflammation. Subcutaneous calcification and fiber-necrosis-forming microinfarctswere reported in anti-NXP2 by Gudipati et al. [4]. The anti-TIF1γ patient displayed typical perifascicular atrophy and perivascular inflammation, while a study reported that perifascicular atrophy with necrotic fibers wasnoted in anti-TIF1γ cases [4].

DM sine dermatitis (DMSD) is an unusual presentation of patients diagnosed with DM, without skin manifestations, occurring in about 8% of patients with DM. In our study, two patients with DMSD were positive for anti-NXP2, one for anti-MDA5, and one for anti-Mi2. In line with previous studies, anti-NXP2 antibodies were significantly associated with DMSD; Inoue et al. found that 86% of DMSD patients were positive for anti-NXP2 [26]. A case report also described DMSD associated with anti-MDA5 antibodies [27]. Notably, skin manifestations may emerge later in the disease course, as observed in one of our patients who developed a V-neck sign and facial erythema after the hospital course.

Patients with DM may present with no evidence of muscle involvement; this subgroup is called amyopathic DM (ADM) and may account for 20% of DM patients [28]. ADM is strongly associated with anti-MDA5 antibodies and ILD [29]. In our cohort, among the three patients diagnosed with ADM, one patient who was positive for anti-MDA5 had interstitial lung disease, while the other two patients showed no pulmonary involvement.

Dysphagia, the most common presentation of esophageal disease in patients with DM, was observed in 59.3% of our patients. This prevalence is higher than the 38.5% reported in Saudi Arabia [18], but lower than the 73.6% noted in Brazil [12]. Moreover, patients with DM have a higher risk of developing malignancy compared to the general population. In our study, six patients had paraneoplastic DM. By comparison, Truzzi et al. reported malignancy in 22% of their cohort, while Le et al. reported a single case of nasopharyngeal carcinoma in 72 patients [12,14]. Anti-TIF1γ antibodies are highly associated with malignancy; the frequency of malignancy occurrence in adult DM patients with anti-TIF1γ ranges from 19 to 100% [10,30]. In our context, two patients with anti-TIF1γ had paraneoplastic DM, one patient with breast cancer and one patient with nasopharyngeal carcinoma, but no significant statistical association was observed between malignancy and anti-TIF1γ. Anti-SAE antibodies, found in 7.4% of our patients, are typically observed in less than 10% of DM cases [22] and have been linked to malignancy in previous studies [18,31]. In our study, one patient with positive anti-SAE antibodies had paraneoplastic DM (breast cancer).

Interstitial lung disease (ILD) was observed in 16.7% of our patients, with dyspnea as the primary symptom. All five patients with anti-MDA5 had ILD, but no significant association was observed between anti-MDA5 and ILD. Patients with anti-MDA5 antibodies have a higher risk of developing ILD and RP-ILD [9,32]. Recently, Lian et al. reported that anti-MDA5-positive DM patients with RP-ILD have a poor prognosis, high mortality, and poor response to treatment [8].

## 5. Conclusions

In this study, we highlighted the clinical manifestations of DM patients and their association with MSAs. This is the first study of the association between clinical features and MSAs in Moroccan patients. The limitation of our study is the small number of patients. Further studies involving larger patient cohorts should be conducted to define additional associations between MSAs and clinical features.

## Figures and Tables

**Table 1:** General characteristics of 54 patients with DM.

Characteristic	Number of Patients (%)
Gender	
Female	40 (74.07%)
Male	14 (25.92%)
Ethnicity	
North African	54 (100%)
Age at diagnosis(in years), mean ± standard deviation	45.8 (±12.95)
Age at onset(in years), mean ± standard deviation	45.6 (±12.93)
Cutaneous manifestations	
V-neck sign	38 (70.4%)
Gottron’s papules	37 (68.5%)
Heliotrope rash	34 (63%)
Periungual erythema	25 (46.3%)
Skin ulcers	15 (27.8%)
Alopecia	13 (24.1%)
Shawl sign	12 (22.2%)
Poikiloderma	6 (11.1%)
Necrotic ulcers	5 (9.3%)
Muscle manifestations	
Myalgia	38 (70.4%)
Proximal muscle weakness	37 (68.5%)
Gastrointestinal manifestations	
Dysphagia	32 (59.3%)
Systemic manifestations	
Arthralgia	26 (48.1%)
Interstitial lung disease	9 (16.7%)
Raynaud’s phenomenon	9 (16.7%)
Malignancy	6 (11.1%)
EMG compatible with DM	42 (97.6%)
Elevated levels of CK and LDH	50 (92.6%)
CK, average (standard error) UI/L	2034 (571.93)
LDH, average (standard error) UI/L	548 (53.61)
Muscle biopsy compatible with DM	11 (64.7%)
Skin biopsy compatible with DM	10 (66.7%)

**Table 2:** Association of MSAs with clinical manifestations.

Manifestation	Anti-TIF1 Gamma		Anti-Mi2		Anti-NXP2		Anti-MDA5		Anti-SAE		Negative	
	N (%)	*p*-Value	N (%)	*p*-Value	N (%)	*p*-Value	N (%)	*p*-Value	N (%)	*p*-Value	N (%)	*p*-Value
Gottron’s papules	9 (90%)	0.214	8 (61.5%)	0.780	2 (40%)	0.167	4 (80%)	0.547	3 (75%)	*	12 (63.2%)	0.532
Heliotrope rash	8 (80%)	0.383	9 (69.2%)	0.836	2 (40%)	0.274	4 (80%)	0.388	4 (100%)	*	9 (47.4%)	0.08
Holster sign	4 (40%)	0.352	3 (23.1%)	1.000	1 (20%)	*	0 (0%)	*	3 (75%)	*	4 (21.1%)	0.416
Shawl sign	4 (40%)	0.203	5 (38.5%)	0.134	0 (0%)	*	0 (0%)	*	1 (25%)	*	2 (10.5%)	0.178
Poïkiloderma	1 (10%)	*	1 (7.7%)	*	1 (20%)	*	0 (0%)	*	1 (25%)	*	2 (10.5%)	1.000
Alopecia	2 (20%)	1.000	5 (38.5%)	0.308	0 (0%)	*	3 (60%)	*	1 (25%)	*	3 (15.8%)	0.294
Periungual Erythema	5 (50%)	1.000	7 (53.8%)	0.531	3 (60%)	0.653	4 (80%)	0.170	2 (50%)	1.000	5 (26.3%)	0.030 *
Skin ulcers	2 (20%)	0.708	6 (46.2%)	0.179	1 (20%)	*	3 (60%)	*	1 (25%)	*	3 (15.8%)	0.147
Necrotic ulcers	2 (20%)	*	0 (0%)	*	0 (0%)	*	3 (60%)	*	0 (0%)	*	1 (5.3%)	0.646
V-neck sign	10 (100%)	0.024 *	9 (69.2%)	1.000	4 (80%)	*	4 (80%)	*	2 (50%)	*	11 (57.9%)	0.139
Proximal muscle weakness	6 (60%)	0.791	10 (76.9%)	0.685	2 (40%)	0.311	3 (60%)	0.645	3 (75%)	*	14 (73.7%)	0.547
Arthralgia	4 (40%)	0.825	4 (30.8%)	0.150	2 (40%)	1.000	4 (80%)	0.184	3 (75%)	0.342	10 (52.6%)	0.627
Myalgia	9 (90%)	0.249	7 (53.8%)	0.251	5 (100%)	*	3 (60%)	*	2 (50%)	*	13 (68.4%)	0.817
Interstitial lung disease	1 (10%)	1.000	2 (15.4%)	1.000	0 (0%)	*	5 (100%)	*	1 (25%)	*	1 (5.3%)	0.203
Raynaud’s phenomenon	2 (20%)	0.667	0 (0%)	0.094	1 (20%)	*	3 (60%)	*	1 (25%)	*	3 (15.8%)	1.000
Dysphagia	7 (70%)	0.682	8 (61.5%)	0.848	4 (80%)	0.638	2 (40%)	0.388	3 (75%)	*	9 (47.4%)	0.190

* Test invalid due to a lack of power.

## Data Availability

The original contributions presented in this study are included in the article. Further inquiries can be directed to the corresponding authors.
